# Innovative App (ExoDont) and Other Conventional Methods to Improve Patient Compliance After Minor Oral Surgical Procedures: Pilot, Nonrandomized, and Prospective Comparative Study

**DOI:** 10.2196/35997

**Published:** 2022-06-28

**Authors:** Deborah Sybil, Meenakshi Krishna, Priyanshu Kumar Shrivastava, Shradha Singh, Imran Khan

**Affiliations:** 1 Department of Oral and Maxillofacial Surgery Faculty of Dentistry Jamia Millia Islamia Delhi India; 2 Faculty of Dentistry Jamia Millia Islamia Delhi India

**Keywords:** ExoDont, mHealth, mobile health, Android app, dental extraction, postoperative, oral surgery, dentistry, teledentistry, mobile app

## Abstract

**Background:**

Postoperative care is influenced by various factors such as compliance, comprehension, retention of instructions, and other unaccounted elements. It is imperative that patients adhere to the instructions and prescribed regimen for smooth and placid healing. ExoDont, an Android-based mobile health app, was designed to ensure a smooth postoperative period for patients after a dental extraction. Besides providing postoperative instructions at defined intervals, the app also sends drug reminders as an added advantage over other available, conventional methods.

**Objective:**

The aim of this study was to compare the compliance rate of individuals with respect to the prescribed regimen and postoperative instructions. Additionally, we aimed to assess any changes in the postoperative complication rate of patients assigned to 3 categories: the verbal, verbal plus written, and ExoDont app-based delivery groups.

**Methods:**

We conducted a pilot, nonrandomized, and prospective comparative study in which patients after tooth extraction were assigned to 3 groups—verbal (Group A), verbal plus written (Group B), and ExoDont app-based delivery (Group C)—based on the eligibility criteria, and a 1-week follow-up was planned to obtain the responses regarding compliance and postoperative complications from the participants.

**Results:**

In total, 90 patients were recruited and equally divided into 3 groups. Compliance to prescribed drug was found to be the highest in Group C, where of the 30 participants, 25 (83%) and 28 (93%) followed the entire course of antibiotics and analgesics, respectively. For postoperative instructions, higher compliance was observed in Group C in relation to compliance to diet restrictions (*P*=.001), not rinsing for 24 hours (*P*<.001), and warm saline rinses after 24 hours (*P*=.001). However, the difference was not significant for smoking restrictions (*P*=.07) and avoiding alcohol (*P*=.16). Moreover, the difference in postoperative complication rate was not statistically significant among the 3 groups (*P=*.31).

**Conclusions:**

As evident from the results, it is anticipated that the ExoDont app will be helpful in circumventing the unaccounted possibilities of missing the prescribed dosage and postoperative instructions and ensuring the smooth recovery of patients after dental extraction. However, future studies are required to establish this app-based method of delivery of postoperative instructions as a viable option in routine clinical practice.

## Introduction

Patient compliance plays an important role in the early and efficient recovery process after dental extraction. It is defined as the degree to which a patient adheres to the prescribed medication, postoperative instructions, self-care, or any therapy sessions given by the doctor. Moreover, the postoperative care period depends on the ability of the patient to comprehend and implement the guidelines as advised by the treating doctor to minimize any surgery-related complications and associated morbidity and improve the quality of life [1].

The lack of adherence to posttreatment guidelines is classified as a major global problem by the World Health Organization [2]. Studies have estimated that around 20%-50% of patients do not take their medication appropriately [3,4]. The reason for this noncompliance could be multifold, including language barriers, low health literacy, inadequate surgeon-patient communication, patient’s inability to concentrate on instructions due to postoperative stress, emotional and psychological state, and other involuntary reasons such as confusion or forgetfulness [5,6]. This clearly demonstrates the need for a system or method to foster adherence in patients and help reduce postoperative complications due to noncompliant issues.

Multiple studies have focused on the methods of dissemination of postoperative instructions after surgery and their influence on the overall quality of treatment. The method of dissemination plays a substantial role in determining the level of postoperative stress and anxiety, pain, postoperative complications, and, most importantly, compliance in patients [7,8]. These studies have compared conventional verbal methods with verbal plus written methods [9], phone-call follow-ups [10], and pictorial methods [8] as viable options for the dissemination of postoperative instructions. However, there is limited literature available for the application of current technology in imparting postoperative instructions after minor oral surgical procedures.

With the advent of the smartphone era and people turning toward mobile apps to accomplish their daily goals, there has been a tremendous growth in technology-based health care delivery systems. In the field of oral surgery, there are multiple apps available that improve access to health care, clinical management, drug guidelines, education, and telecommunication, etc. Smartphone apps such as iResus, BNF, and Snellen are available for these purposes [11]. Based on similar principles, the ExoDont app was developed to ensure compliance in patients for prescribed drug regimen and postoperative instructions dissemination. ExoDont is an Android-based hybrid app aimed at fostering treatment adherence in patients undergoing dental extractions. It is an attempt toward making the public more self-reliant regarding their prescribed medication dosage, frequency, and duration with a personalized, easy-to-use, and innovative app-based system that displays reminders at the appropriate times for taking medication and illustrates postoperative instructions.

As the first part to the project, the development of the ExoDont app, its feasibility, functionality, and preliminary field-testing results were presented in a previous report [12]. As a second part, this study described a detailed comparison of conventional (verbal and verbal plus written) and ExoDont app-based methods of postoperative instructions dissemination. The primary objective of this study was to evaluate and compare patients’ compliance to the postoperative instructions and prescribed drug regimen with respect to the 3 groups of dissemination methods: verbal, verbal plus written, and app-based. As a secondary objective, the complication rate after tooth extraction was evaluated and compared for the 3 groups.

## Methods

### Study Design

This was a pilot, nonrandomized, and prospective comparative study carried out in the outpatient department of oral and maxillofacial surgery of a tertiary dental care institute. All study participants were well-informed about the study, and written informed consent was obtained. A sample size of 90 patients was recruited, and the patients were assigned to 3 different groups based on the methods of dissemination of postoperative instructions as per the eligibility criteria. The first group, labeled “Group A,” received only verbal instructions; the second group, labeled “Group B,” received both verbal and written instructions in the form of a pamphlet; and the third group, labeled “Group C,” received ExoDont app-based postoperative instructions and medication reminders.

### Ethics Approval

This study was conducted at a tertiary dental care institute at the Faculty of Dentistry, Jamia Millia Islamia, Delhi over a period of 3 months. Ethical approval was obtained from the Institutional Ethical Committee of the Faculty of Dentistry, Jamia Millia Islamia, Delhi, under proposal number 2(1/10/291/JMI/IEC/2020). Informed consent was obtained from the participants for being a part of the study.

### Postoperative Instructions and Treatment Protocol

**Figure 1:**
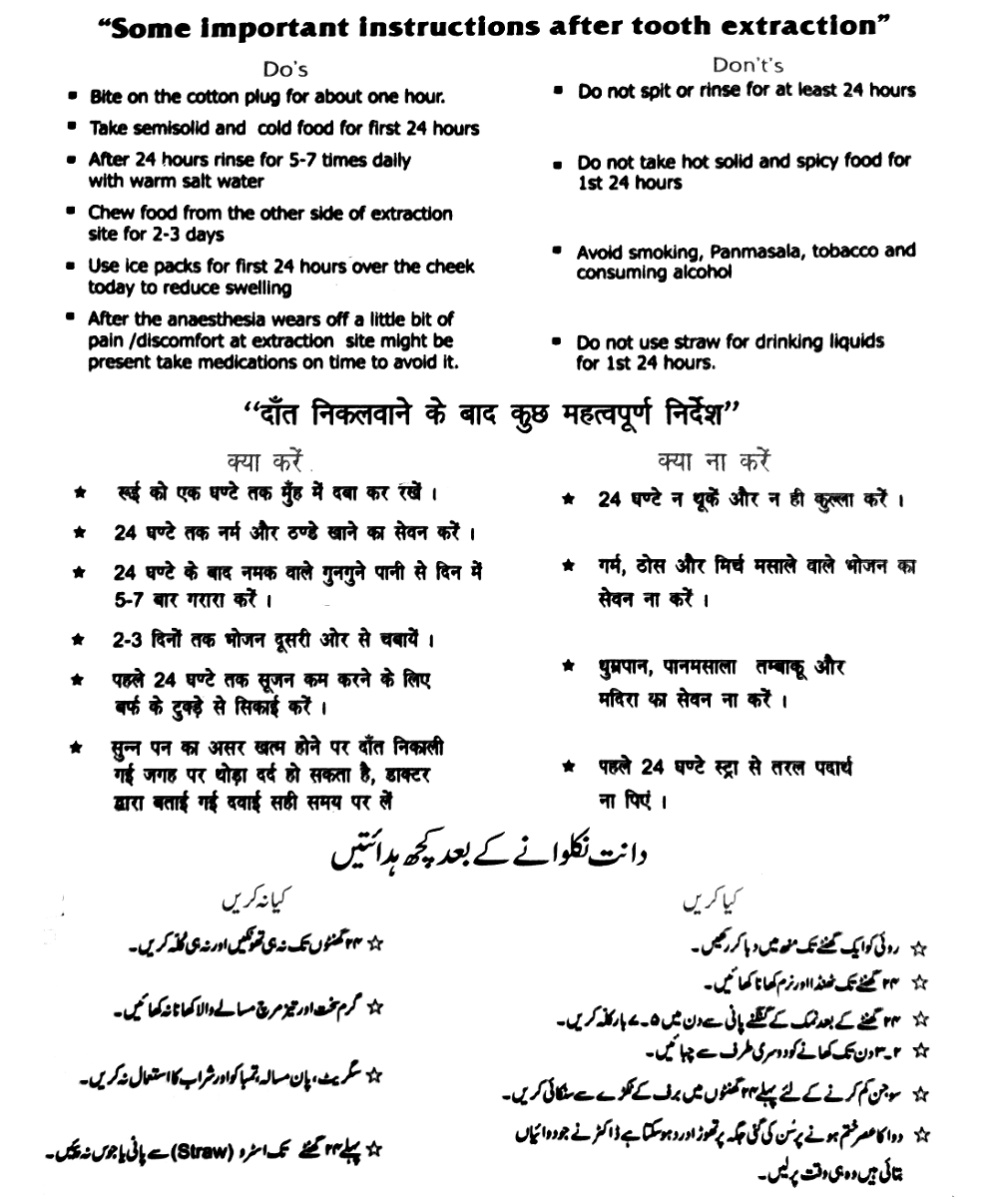
Written postoperative instructions in the form of a pamphlet for Group B patients.

**Figure 2:**
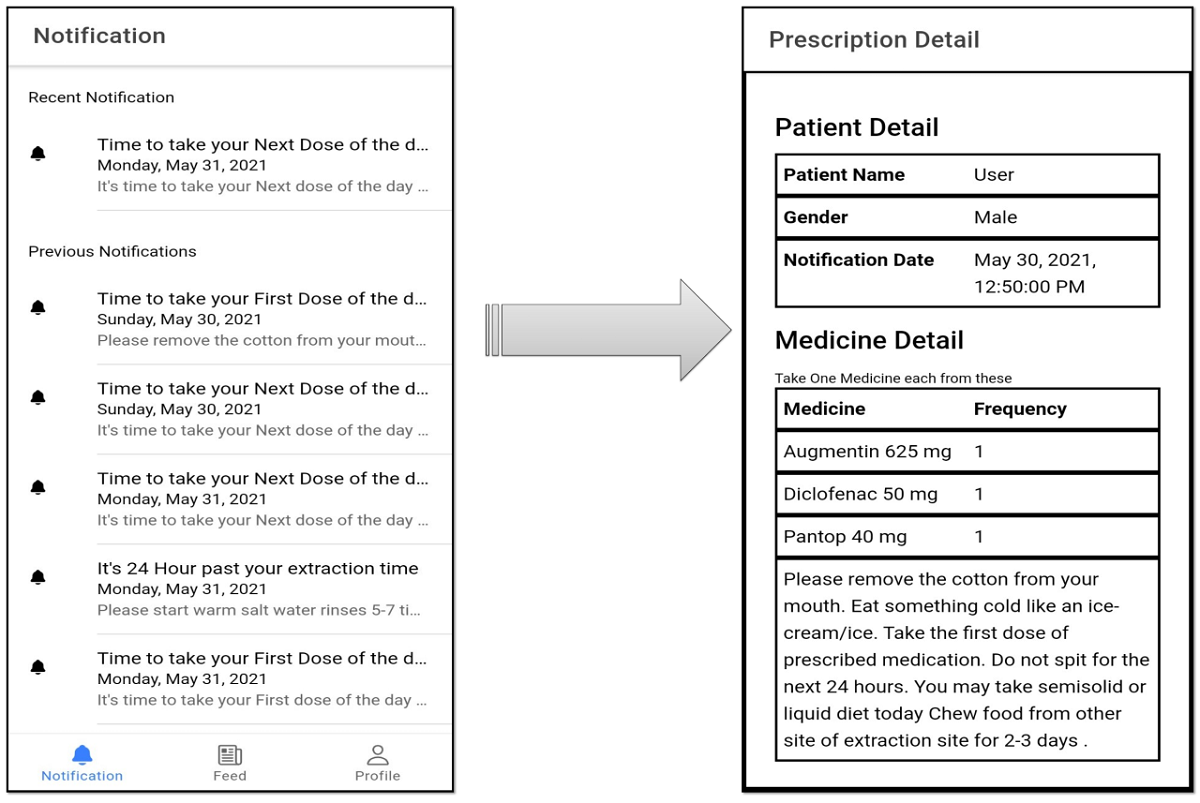
ExoDont app-based postoperative instructions in the form of notifications for Group C patients.

The postoperative treatment protocol was established, comprising antibiotics and analgesics along with postextraction instructions to be given to patients after tooth extraction. A list of common postoperative instructions was formulated by referring to previously existing data [1]. The same postextraction instructions were then disseminated through the 3 methods: in the form of verbal instructions to Group A patients; verbal as well as written instructions through a well-designed pamphlet in the 3 languages of English, Hindi, and Urdu (Figure 1) to Group B patients; and through the ExoDont app to Group C patients (Figure 2).

### Workflow of the ExoDont App

ExoDont is a notification-based Android app, which sends out pop-ups reminding patients about their prescribed dosage of medicine and the postoperative instructions to follow. The ExoDont app has been described in detail in a previous study [12].

The ExoDont app supports 2 platforms—1 for the surgeon and administrative staff and 1 for the patient. The administrative staff enters the patients’ details such as name, age, gender, time and date of the procedure, and phone number on the ExoDont webpage. Subsequently, the administrative staff has to choose from the list of options the names of the antibiotics and analgesics to be used, duration for each of these drugs, and frequency for which the patient has to take the prescribed drug. The options for the duration of the drug regimen are available as short course (3 days), standard course (5 days), and long course (7 days).

After the tooth extraction, patients have to download the app on their smartphone from the Google Play Store. Patients receive an introductory message as soon as they log on the app. This message displays the demographic details of the patient and the date and time of the procedure as entered by the administrative staff. Based on the time of the procedure, patients would receive scheduled reminders for postoperative instructions and prescribed drug at defined intervals as shown in Table 1.

**Table 1:** Content of ExoDont notifications for postoperative instructions and drug reminders.

Time from procedure	Message content
1 hour	Remove the ice pack Eat something soft and cold such as ice cream Take the first dose of antibiotics and analgesics
8-12 hours (depending on the drug chosen by the administrative staff)	Take the second dose of antibiotics and analgesics Avoid hot and hard food Chew only on the opposite side
16-24 hours (depending on the drug chosen by the administrative staff)	Take the third dose of antibiotics and analgesics Start warm saline rinses 4-5 times daily Brush regularly

### Patient Inclusion and Exclusion Criteria

Adult patients (aged >18 years) undergoing routine tooth extraction at the department of oral and maxillofacial surgery were included in the study. Patients with psychological disorders or mental conditions that created difficulties in language comprehension and those with medical conditions that made them prone to postoperative complications were not included in the study. Additionally, patients who could not read the instruction pamphlet (for Group B) and those who did not have a smartphone (for Group C) were not included in either of the 2 groups; however, they were included in Group A. Recruitment continued as per the eligibility criteria until the target of 30 participants for each group was reached.

### Procedure

Simple intra-alveolar extraction was performed by the treating surgeon in the outpatient department of oral and maxillofacial surgery. After the extraction, patients were given a written prescription for the drugs to be taken. Subsequently, postoperative instructions were given to each patient verbally by the surgeon. Patients in Group A received only verbal instructions in 1 of the 3 languages—Hindi, Urdu, or English—whichever they found convenient. The patients in Group B received a pamphlet containing written postoperative instructions, printed in all 3 languages—Hindi, Urdu, and English—along with verbal instructions. Lastly, patients in Group C were asked to download the free ExoDont app from the Google Play Store on their smartphones, through which they received reminders for postoperative instructions and drug intake.

### Data Collection

Each patient was called to the outpatient department of oral and maxillofacial surgery for follow-up 1 week after the extraction, and their responses to a group-specific feedback form were obtained (Multimedia Appendix 1). The patients who could not report after 1 week were followed up through a phone call. In the group-specific feedback form that was administered by the investigators, yes-or-no questions for each drug taken and postoperative instruction followed were asked separately. The criteria for compliance measurement were set at 100% of the prescribed dosage and postoperative instructions followed by the patients, since the course duration of 3 to 7 days was very short. All the complications were reported directly by patients during their follow-up and assessed by the treating surgeon according to the standard criteria of evaluation.

### Data Analysis

The analysis of data collected in response to the feedback form was done using SPSS statistical package (version 10.0; IBM Corp). Chi-square test was applied to compare the rates of compliance and complication in the 3 groups. The level of significance was set at *P*<.05.

## Results

### Participant Demographics

A total of 90 patients were recruited with 30 participants in each group. The mean age of the patients who participated in the study was 40.4 (range 25-56) years for Group A, 36.2 (range 21-52) years for Group B, and 34.3 (range 19-47) years for Group C. Of the 90 patients who participated in the study, 53 (59%) were men and 37 (41%) were women. Among each group of 30 patients, 18 (60%) men and 12 (40%) women were in Group A; 19 (63%) men and 11 (37%) women were in Group B; and 16 (53%) men and 14 (47%) women were in Group C. The most common teeth to be extracted were mandibular molars, followed by the maxillary molars.

### Comparative Evaluation of Groups A (Verbal), B (Verbal Plus Written), and C (App-Based)

The chi-square test revealed higher compliance rate in Group C to antibiotics (*P*<.001), analgesics (*P*<.001), diet restrictions (*P*=.001), not rinsing for 24 hours (*P*<.001), and warm saline rinses after 24 hours (*P*=.001). However, no significant differences were found among the 3 groups for postoperative instructions relating to smoking restrictions (*P*=.07) and avoiding alcohol (*P*=.16). A detailed analysis of the comparison has been presented in Table 2. Some of the postoperative complications observed in all 3 groups were bleeding, dry socket, infection, and pain. A majority (82%, 74/90) of the participants did not experience any complications. The difference observed in the 3 groups for the rate of postoperative complications was not significant (*P*=.31; Table 3).

**Table 2:** Comparison of compliance in Group A (verbal), Group B (verbal plus written), and Group C (app-based).

Compliance	Group A (n=30), n (%)	Group B (n=30), n (%)	Group C (n=30), n (%)	*P* value
Antibiotics	10 (33)	13 (43)	25 (83)	<.001
Analgesics	13 (43)	10 (33)	28 (93)	<.001
Diet restrictions	22 (73)	15 (50)	28 (93)	.001
Not rinsing for 24 hours	10 (33)	20 (67)	26 (87)	<.001
Smoking restrictions	20 (67)	25 (83)	27 (90)	.07
Avoiding alcohol	24 (80)	20 (67)	26 (87)	.16
Warm saline rinses	8 (27)	14 (47)	23 (77)	.001

**Table 3:** Comparison of postoperative complications in Group A (verbal), Group B (verbal plus written), and Group C (app-based).

		Group A (n=30), n (%)	Group B (n=30), n (%)	Group C (n=30), n (%)	*P* value	
**Complications**						.31
	Bleeding	2 (7)	0 (0)	1 (3)		
	Dry socket	5 (17)	5 (17)	1 (3)		
	Infection	1 (3)	0 (0)	0 (0)		
	Pain	0 (0)	0 (0)	1 (3)		
	None	22 (73)	25 (83)	27 (90)		

### Intergroup Comparison

Comparison between Groups A and B revealed no significant differences between the 2 groups in relation to the compliance to antibiotics (*P*=.43) and analgesics (*P*=.43); adherence to postoperative instructions such as compliance to diet restrictions (*P*=.06), warm saline rinses (*P*=.11), smoking restrictions (*P*=.14), and alcohol restrictions (*P*=.24); or the rate of postoperative complications (*P*=.35). However, higher compliance to not rinsing for 24 hours (*P*=.01) was observed in Group B.

When compared to Group A, Group C had higher compliance to antibiotics (*P*<.001) and analgesics (*P*<.001). Most postoperative instructions such as diet restrictions (*P*=.04), not rinsing for 24 hours (*P*<.001), warm saline rinses (*P*<.001), and smoking restrictions (*P*=.03) also showed higher compliance in the app-based group. However, the difference was not significant for alcohol restrictions during the postoperative phase (*P*=.49) and the rate of postoperative complications (*P*=.10).

Similarly, the comparison between the verbal plus written (Group B) and app-based delivery (Group C) groups showed significant differences for compliance to drug regimen (antibiotics: *P*=.001; analgesics: *P*<.001) and postoperative instructions such as compliance to diet restrictions (*P*<.001) and warm saline rinses (*P*=.02). However, the differences were not significant for compliance to not rinsing for 24 hours (*P*=.07), smoking restrictions (*P*=.14), and alcohol restrictions (*P*=.07) and the rate of postoperative complications (*P*=.71).

### Feedback for the Written Instructions

According to the responses from patients in Group B, 23 (77%) out of 30 participants found the pamphlet for written instructions useful. Second, most (15/30, 50%) of the patients referred to the pamphlet only once, followed by 0 times (9/30, 30%; Table 4).

**Table 4:** Feedback for the written instructions.

Question, response		Patient (n=30), n (%)
**Was the pamphlet with written instructions helpful?**		
	Yes	23 (77)
	No	7 (23)
**How many times did you refer to the pamphlet during the week?**		
	0	9 (30)
	1	15 (50)
	2	4 (13)
	3	1 (3)
	>3	1 (3)

### Feedback for the ExoDont App-Based Delivery of Instructions

Based on the responses of Group C patients from the feedback form, the ExoDont app was found useful by 83% (25/30) of the patients. However, 13% (4/30) of the users found it inconvenient due to the ExoDont app’s requirement of an active internet connection throughout the duration of its use. Furthermore, a small number of patients did not receive timely notifications due to some technical issues that might have occurred during the entry of data or an unstable internet connection (Table 5). It was reported that the ExoDont app helped the most in adherence to the drug regimen (70%, 21/30), followed by postoperative instructions for diet restrictions (67%, 20/30) and warm saline rinses (63%, 19/30; Table 5).

**Table 5:** Feedback for the ExoDont app-based delivery of instructions.

Question, response			Patient (n=30), n (%)
**Did you find the ExoDont app useful?**			
	Yes	25 (83)	
	No	5 (17)	
**Did the ExoDont app cause any inconvenience?**			
	Yes	4 (13)	
	No	26 (87)	
**Which of the following instructions did the app help you with?**			
	Taking medication	21 (70)	
	Ice pack and semisolid diet in the first 24 hours	20 (67)	
	Warm saline rinses	19 (63)	
	Avoiding smoking and alcohol	16 (53)	

## Discussion

### Principal Findings

In this study, Group A patients were found the least compliant for their prescribed dosage of antibiotics, whereas Group C showed a higher rate of compliance than the other groups (*P*<.001). Additionally, a higher compliance rate for the analgesics prescription (*P*<.001) was observed in Group C. A similar trend was observed in adherence to postoperative instructions. Intergroup comparison also produced results in favor of the app-based delivery system, followed by the verbal plus written method of dissemination of postoperative instructions. The ExoDont app-based system was able to accomplish its goal of improving patient compliance after a dental extraction. Although the compliance rates in Group C toward drugs and postoperative instructions were found to be higher, the app did not seem to have any prominent effect on the reduction of complication rates. This can be attributed to the fact that the complications following dental extraction depend on a number of variables—alteration of any or none of which may have an impact on the occurrence of complications. These factors include the technique used by the surgeon, patient characteristics, severity of trauma to the tissue, any underlying health conditions, and the number and type of teeth extracted [13].

Verbal postoperative instructions given to patients are a part of routine postoperative care. However, compliance to this conventional method varies. Blinder et al [9] studied patient compliance to postoperative instructions after oral surgical procedures in 3 groups—verbal, written, and verbal plus written instructions—where the highest compliance (60%) was observed in the verbal plus written group, followed by 36% in the written group and only 4% in the verbal group. This can be attributed to the patient’s mental capacity to retain information, which is different for every patient. In this study, of the 30 participants in Group B, 9 (30%) did not refer to the written instructions through a pamphlet at all, and 15 (50%) only referred to them once, which signifies patient unacceptability toward written medical information. A previous study by Alvira-González et al [1] hypothesized that patients would remember information if the mechanism behind it were explained to them. Therefore, additional written information was given to the third group in addition to the verbal and written methods. However, no major difference in the adherence to postoperative instructions was found regarding the manner of the presentation of instructions in this study. This again raises concerns about the practicality of presenting written information as postoperative instructions to patients. Other elements such as phone call follow-ups [10] and visual graphics [8] have also been used for disseminating postoperative instructions, which achieved better results than conventional verbal and written practices.

Multiple studies have discussed the importance of the delivery method of postoperative instructions in the postoperative care of patients [1,8-10]. The importance of postoperative instructions cannot be overemphasized in the adequate healing of the socket and soft tissues after dental extraction. Failure to adhere to postoperative instructions and medication prescription may lead to delayed healing and increased risk of postoperative complications, adding to physical and emotional stress and increasing monetary expenditure to patients. Some of the commonly observed postextraction complications are dry socket or alveolar osteitis, which can cause considerable pain to an individual; postoperative infection at the site of surgery; postextraction hemorrhages; and paresthesia [1]. A positive correlation between the occurrence of alveolar osteitis and lack of compliance toward instructions such as using mouthwash or refraining from smoking has been established previously [14].

The field of perioperative management has seen huge technological advancements in recent times, advocating the use of smartphone apps to ensure a stress-free and easy recovery of the patient. In this regard, some of the apps available are “Teen Pocket PATH” [15] for medication adherence, “Panda” app [16] for postoperative pain management, and “Buddy Healthcare” [17]—a broad platform that provides preoperative assessment; reminders for patients as to when to stop eating and drinking and start taking medications; instructions for physiotherapy exercises; wound care instructions; a list of medications after surgery; and monitoring of recovery progress after surgery by care personnel. Other mobile apps being used for medication adherence are “RxmindMe” [18], which informs patients when the dose is due and additionally has a provision for recording when the dose was taken; and “SmartTrack” [19], which sends alerts if patients miss a dose for inhaler devices. The ExoDont app differs from these available systems in that it delivers specific postoperative instructions designed for patients undergoing dental extraction and drug reminders for medication adherence. The requirement of an uninterrupted internet connection is primarily needed when several mobile health (mHealth) apps and devices are being created and promoted each day. ExoDont, similar to other mHealth apps, requires an internet connection for sending timely notifications, which could also be listed as its drawback.

The role of preoperative anxiety and stress as an aggravating factor for postoperative pain has been well-established [20]. To foster compliance and reduce complications in patients, the dissemination methods of instructions could be modified to include newer and innovative techniques that reduce the burden on patients to remember numerous instructions and alleviate the anxiety factor in patients anticipating surgery, thus minimizing postoperative pain. Current evidence in postoperative management has been in favor of technology-based health care delivery systems. The use of mobile apps in postoperative care has been found to reduce a substantial amount of time, travel, and cost to patients along with a higher satisfaction level for both patients and providers [21,22]. Therefore, in an attempt to provide a well-optimized postoperative recovery period, the innovative mobile app ExoDont was introduced. The notification-based ExoDont app enables benefit for users of all ages because it does not require any manual inputs from the patients. The ExoDont app presents a user-friendly environment, even for those who are not adept with the use of smartphones. The results obtained from this study suggest that the ExoDont app had been well-received by a group of patients and, therefore, can be used in the future for the advancement of technology in the field of perioperative management.

There are, however, a few limitations to this study that need to be discussed. First, there was a lack of homogeneity among the 3 groups in the selection of patients for the type and number of the teeth to be extracted, which could have altered the rate of complications assessed. However, since a majority of the teeth to be extracted were mandibular molars, the differences have not been substantial. The selection of a specific type and number of teeth to be extracted could be considered in future studies to test the validity of the app. Second, the study does not take into account the educational level or health literacy of the patients, which can influence the perception of individuals using an mHealth app such as ExoDont. The comparison was drawn from a subset of the population. Future studies could include a larger sample size by using a multicentric approach to test the app across different centers with varying literacy levels. Third, since this was a pilot study, a nonrandomized design was used to enroll patients into the 3 groups, with different eligibility criteria for each group. This approach does not generate conclusive evidence for comparison among the 3 groups. Therefore, more randomized trials are required to accurately evaluate the benefits of the ExoDont app over conventional methods and determine the difference in the postextraction rate of complications among the groups.

### Conclusion

This study promotes the use of technology to ensure the smooth and efficient postoperative recovery of patients after minor oral surgical procedures. As established through this study, the ExoDont app succeeded in fostering compliance in patients to the prescribed drug regimen and adherence to postoperative instructions in a subset of the population. This study encourages the use of the app-based delivery method of postoperative instructions over conventional verbal and written methods, which could play a beneficial role in bridging the gap between the surgeon and patient and improve compliance in patients. Although the app has been found to be effective in this study, more randomized studies are required to establish the advantage of this app-based dissemination method of instructions over other conventional techniques.

## Appendix

Multimedia Appendix 1 Group-specific feedback forms for Groups A, B, and C.

## Abbreviations

mHealth: mobile health

## Data Availability

The data sets generated during and/or analyzed during the current study are available from the corresponding author on reasonable request.

## References

[1] Alvira-González Joaquín, Gay-Escoda C (2015). Compliance of postoperative instructions following the surgical extraction of impacted lower third molars: a randomized clinical trial. Med Oral Patol Oral Cir Bucal.

[2] (2003). Adherence to long-term therapies : evidence for action. World Health Organization.

[3] Brown MT, Bussell JK (2011). Medication adherence: WHO cares?. Mayo Clin Proc.

[4] Bosworth HB, Zullig LL, Mendys P, Ho M, Trygstad T, Granger C, Oakes MM, Granger BB (2016). Health information technology: meaningful use and next steps to improving electronic facilitation of medication adherence. JMIR Med Inform.

[5] Panesar K (2012). Medication management: patient compliance and health behavior models. US Pharmacist.

[6] Kessels RPC (2003). Patients' memory for medical information. J R Soc Med.

[7] Gheisari R, Resalati F, Mahmoudi S, Golkari A, Mosaddad SA (2018). Do different modes of delivering postoperative instructions to patients help reduce the side effects of tooth extraction? a randomized clinical trial. J Oral Maxillofac Surg.

[8] Shenoi RS, Rajguru JG, Parate SR, Ingole PD, Khandaitkar SR, Karmarkar JS (2021). Compliance of postoperative instructions following the surgical extraction of impacted lower third molars. Indian J Dent Res.

[9] Blinder D, Rotenberg L, Peleg M, Taicher S (2001). Patient compliance to instructions after oral surgical procedures. Int J Oral Maxillofac Surg.

[10] Aloy-Prósper Amparo, Pellicer-Chover H, Balaguer-Martínez José, Llamas-Monteagudo O, Peñarrocha-Diago Miguel (2020). Patient compliance to postoperative instructions after third molar surgery comparing traditional verbally and written form versus the effect of a postoperative phone call follow-up a: a randomized clinical study. J Clin Exp Dent.

[11] Carey E, Payne KFB, Ahmed N, Goodson A (2015). The benefit of the smartphone in oral and maxillofacial surgery: smartphone use among maxillofacial surgery trainees and iPhone apps for the maxillofacial surgeon. J Maxillofac Oral Surg.

[12] Krishna M, Sybil D, Shrivastava PK, Premchandani S, Kumar H, Kumar P (2021). An innovative app (ExoDont) for postoperative care of patients after tooth extraction: prototype development and testing study. JMIR Perioper Med.

[13] Bui CH, Seldin EB, Dodson TB (2003). Types, frequencies, and risk factors for complications after third molar extraction. J Oral Maxillofac Surg.

[14] Vettori E, Costantinides F, Nicolin V, Rizzo R, Perinetti G, Maglione M, Di Lenarda R (2019). Factors influencing the onset of intra- and post- operative complications following tooth exodontia: retrospective survey on 1701 patients. Antibiotics (Basel).

[15] Shellmer DA, Dew MA, Mazariegos G, DeVito Dabbs A (2016). Development and field testing of Teen Pocket PATH(®), a mobile health application to improve medication adherence in adolescent solid organ recipients. Pediatr Transplant.

[16] Dunsmuir D, Wu H, Sun T, West NC, Lauder GR, Görges Matthias, Ansermino JM (2019). A postoperative pain management mobile app (Panda) for children at home after discharge: usability and feasibility. JMIR Perioper Med.

[17] Buddy Healthcare.

[18] Aungst T (2012). Rxmind Me app review, a simple medication reminding tool for patients. iMedicalApps.

[19] Foster JM, Usherwood T, Smith L, Sawyer SM, Xuan W, Rand CS, Reddel HK (2014). Inhaler reminders improve adherence with controller treatment in primary care patients with asthma. J Allergy Clin Immunol.

[20] Robleda G, Sillero-Sillero A, Puig T, Gich I, Baños Josep-E (2014). Influence of preoperative emotional state on postoperative pain following orthopedic and trauma surgery. Rev Lat Am Enfermagem.

[21] Semple JL, Sharpe S, Murnaghan ML, Theodoropoulos J, Metcalfe KA (2015). Using a mobile app for monitoring post-operative quality of recovery of patients at home: a feasibility study. JMIR mHealth uHealth.

[22] Gunter RL, Chouinard S, Fernandes-Taylor S, Wiseman JT, Clarkson S, Bennett K, Greenberg CC, Kent KC (2016). Current use of telemedicine for post-discharge surgical care: a systematic review. J Am Coll Surg.

