# Supplementary material for: Innovative App (ExoDont) and Other Conventional Methods to Improve Patient Compliance After Minor Oral Surgical Procedures: Pilot, Nonrandomized, and Prospective Comparative Study
Source: JMIR Perioper Med. 2022 Jun 28;5(1):e35997. doi: 10.2196/35997 (PMC9277528; doi:10.2196/35997)
Supplement: Multimedia Appendix 1 [file periop_v5i1e35997_app1.docx]

**SUPPLEMENTARY FILE**

**FEEDBACK FORM FOR 1 WEEK_FOLLOW-UP FOR GROUP A, B, and GROUP C PATIENTS**

**Group-A**

**Reference no- Name-**

**Age/Sex- Occupation-**

1- Did you strictly follow the antibiotic medication prescribed?

Yes / No…...If not, how many days did you follow?..........

2- Did you strictly follow the NSAID medication prescribed?

Yes / No…...If not, how many days did you follow?..........

3- Did you strictly follow the recommendation of maintaining a soft-temperate diet for 24 hours?

Yes / No

4 - Did you strictly follow the recommendation of not rinsing or spitting for the first 24 hours after surgery?

Yes / No

5 - Concerning smokers, did you strictly follow the recommendation of not smoking for seven days? Yes / No…...If not, how many days did you follow?........

6- Did you strictly follow the recommendation of not drinking alcoholic/carbonated beverages for the following seven days?

Yes / No…...If not, how many days did you follow?........–

7- Did you strictly follow the recommendation of warm saline rinses during the week?

Yes / No…...If not, how many days did you follow?.....

**Group-B**

**Reference no- Name –**

**Age/sex- Occupation-**

1- Did you strictly follow the antibiotic medication prescribed?

Yes / No…...If not, how many days did you follow?..........

2- Did you strictly follow the NSAID medication prescribed?

Yes / No…...If not, how many days did you follow?..........

3- Did you strictly follow the recommendation of maintaining a soft-temperate diet for 24 hours?

Yes / No

4 - Did you strictly follow the recommendation of not rinsing or spitting for the first 24 hours after surgery?

Yes / No

5 - Concerning smokers, Did you strictly follow the recommendation of not smoking for seven days? Yes / No…...If not, how many days did you follow?........

6- Did you strictly follow the recommendation of not drinking alcoholic/carbonated beverages for the following seven days?

Yes / No…...If not, how many days did you follow?........ –

7- Did you strictly follow the recommendation of warm saline rinses during the week?

Yes / No…...If not, how many days did you follow?.....

8. Was the pamphlet with written instructions helpful? Yes/ No..if No, why?..........................

9. How many times did you refer to pamphlet during the week?

a) Zero b)one c)two d)three e)more than three

**Group-C**

**Reference no- Name-**

**Occupation - age/sex-**

1- Did you strictly follow the antibiotic medication prescribed?

Yes / No…...If not, how many days did you follow?..........

2- Did you strictly follow the NSAID medication prescribed?

Yes / No…...If not, how many days did you follow?..........

3- Did you strictly follow the recommendation of maintaining a soft-temperate diet for 24 hours? Yes / No

4 - Did you strictly follow the recommendation of not rinsing or spitting for the first 24 hours after surgery? Yes / No

5 - Concerning smokers, Did you strictly follow the recommendation of not smoking for seven days? Yes / No…...If not, how many days did you follow?........

6- Did you strictly follow the recommendation of not drinking alcoholic/carbonated beverages for the following seven days?

Yes / No…...If not, how many days did you follow?........ –

7- Did you strictly follow the recommendation of warm saline rinses during the week?

Yes / No…...If not, how many days did you follow?.....

8. Did you find the app helpful? Yes/No

9. Which of the following instructions did the app help you with?

a)Taking medication

b) Ice pack and semi-solid diet in first 24hr.

c) Warm salt water rinses

d)Avoid smoking and alcohol

10. Did the app cause any inconvenience

a)Yes b) No if yes, What --------------------------------------

11. Any suggestion on improving the app…………..
